# Stability Analysis of Polymer Flooding-Produced Liquid in Oilfields Based on Molecular Dynamics Simulation

**DOI:** 10.3390/ma18102349

**Published:** 2025-05-18

**Authors:** Qian Huang, Mingming Shen, Lingyan Mu, Yuan Tian, Huirong Huang, Xueyuan Long

**Affiliations:** 1 School of Petroleum Engineering, Chongqing University of Science and Technology, Chongqing 401331, China; huangqianswpu@163.com (Q.H.); swpu_tianyuan@126.com (Y.T.); hryh@cqust.edu.cn (H.H.); 2 Chongqing Gas Group Co., Ltd., Nan’an Branch, Chongqing 400060, China; 3 School of Safety Science and Engineering, Chongqing University of Science and Technology, Chongqing 401331, China

**Keywords:** polymer, oil–water emulsion, molecular dynamics, stability

## Abstract

The S oilfield has adopted polymer flooding technology, specifically using partially hydrolyzed polyacrylamide (HPAM), to enhance oil recovery. During the production process, the S oilfield has generated a substantial amount of stable polymer flooding-produced liquid, in which oil droplets are difficult to effectively coalesce, presenting significant challenges in demulsification. This article focuses on the produced fluids from S Oilfield as the research subject, developing a molecular dynamics model for the stability analysis of production liquid, including the molecular dynamics model of an oil–pure water system, an oil–mineralized water system and an oil–polymer–mineralized water system, using the principle of molecular dynamics and combining it with the basic molecular model for analyzing the stability of polymer flooding-production liquid. Through the molecular dynamics simulation of the stability analysis of the extracted liquid, the changing rules of the molecular diffusion coefficient, radial distribution function (RDF), interfacial interaction energy, and interfacial tension under the action of ions as well as polymers in water were investigated. The simulation results demonstrate that the presence of all three inorganic salt ions (Na^+^, Ca^2+^, and Mg^2+^) reduces the interfacial tension between oil and water and stabilizes the interface. Following the addition of polymer, the interfacial tension of the system decreases and the interfacial interaction energy increases significantly, indicating that the stability of the system is significantly enhanced by HPAM.

## 1. Introduction

Heavy oil is mainly composed of alkanes, aromatic hydrocarbons, resins and asphaltenes, and contains heteroatoms such as N, O and S [1]. Due to its high content of asphaltenes and resins, compared with conventional crude oil, heavy oil is characterized by high density and high viscosity, which increases the difficulty of extraction [2]. As crude oil extraction advances, traditional oilfields in China have reached a stage of high moisture content [3]. In order to further improve crude oil recovery, stabilize crude oil production and control moisture content, tertiary enhanced oil recovery (EOR) technology has been rapidly developed [4]. Chemical flooding technology has been widely used in tertiary oil recovery, with polymer flooding being relatively mature and highly effective [5]. During polymer flooding for oil recovery, a large number of oil-in-water (O/W) emulsions are generated [6]. In these emulsions, heavy oil acts as the dispersed phase, while water serves as the continuous phase. At present, partially hydrolyzed polyacrylamide (HPAM) is the polymer most frequently utilized in China [7]. Due to the effect of the polymer at the oil–water interface, the stability of the emulsion is significantly influenced. This results in the inability of oil droplets to efficiently coalesce, making emulsion breaking highly challenging. Consequently, the efficiency of oil–water separation is low, which directly impacts subsequent ground operations, including further production, processing, and the treatment of oily wastewater [8,9].

For oil–water emulsion stability studies, the main focus is on oil–water emulsion interfacial behavior studies [10]. Common methods for studying oil–water emulsion interfacial behavior include experimental approaches and simulation techniques [11]. The experimental method has the advantage of visualization of the results, and many scholars at home and abroad have carried out a large number of experimental studies on emulsion stability, and also produced a number of related experimental results that have been widely recognized. Goual et al. [12] described interfacial films stabilizing oil–water emulsions on the nanoscale using transmission electron microscopy (TEM) techniques. TEM micrographs reveal that the film maintaining interfacial stability consists of vertically stacked worm-like asphaltene clusters. These clusters are formed by the aggregation of highly interfacially active asphaltene molecules. They significantly reduce oil–water interfacial tension, thereby enhancing emulsion stability. Liu et al. [13] performed emulsion stability experiments using droplet shape analysis and conductivity measurements. These experiments aimed to study the effects of interactions between asphaltenes and colloids on oil–water interfacial properties and emulsion stability. Ese et al. [14] investigated the influence of naphthenic acids and their salts on emulsion stability through a series of experiments. The results demonstrated that, under specific conditions, naphthenic acids and their salts significantly enhance the stability of W/O emulsions. However, experimental studies on the interfacial behavior of oil–water emulsions are often limited to the macroscopic level. This limitation hinders a deeper understanding of the stability mechanisms of oil–water emulsions. Mohamed et al. [15] employed the hydrophilic–lipophilic balance (HLB) of surfactants to examine the influence of surfactant structure on the stability of W/O emulsions. The study revealed that the amine acetate series demonstrated greater stability at lower concentrations than the glycol ether series of surfactants.

With the rapid advancements in computer technology, molecular simulation has become an emerging computerized chemistry technology, which, by virtue of its powerful computational capability, can quickly obtain the results of complex intermolecular calculations and reveal the relationship between structure and properties at the microscopic level. Among these, molecular dynamics (MD) is one of the more mainstream molecular simulation methods, which can effectively make up for the shortcomings of experimental methods, and can study the properties of oil–water interface in emulsions from the microscopic level, providing theoretical support for further understanding of the stability mechanism of oil–water emulsions [16].

Rogel et al. [17] investigated the aggregation behavior of asphaltene molecules in a vacuum environment using the MD method and calculated the solubility parameters of two asphaltene average structures and their aggregates. The results showed that the solubility parameter of asphaltene aggregates decreased significantly as the number of asphaltene aggregates increased. In addition, they found that the stability of asphaltene dimers increased with the increase in the heptane/toluene volume ratio. Liu et al. [18] investigated the role of asphaltenes in stabilizing oil–water emulsions using MD simulation. A variety of asphaltene structural models were selected for simulation and it was found that asphaltenes effectively stabilize the emulsion system by forming an interfacial film with significant mechanical strength at the oil–water interface through self-polymerization. Lan et al. [19] investigated the effect of graphene oxide (GO) on the stability of O/W and W/O type emulsions in the presence of violanthrone-79 (VO-79) in asphaltene by MD simulations. The results showed that GO first formed a film around the emulsion droplets and then enhanced the adhesion between droplets or between droplets and the macroscopic oil/water interface through film-to-membrane interactions, thereby destabilizing the W/O or O/W emulsions. Sun et al. [20] investigated the interfacial stabilization mechanism of asphaltene-rich O/W emulsions formed by alkali-surfactant-polymer (ASP) flooding-produced water using molecular dynamics (MD) simulations. The results demonstrated that the interfacial formation energy of the composite asphaltene-sodium dodecyl benzene sulfonate (SDBS) film system was significantly higher than that of the single-layer SDBS system. This enhanced interfacial film stability was attributed to the cross-linked aggregation of asphaltene and SDBS molecules at the oil/water interface.

At present, molecular simulation has been widely applied in the study of the interfacial properties of surfactant-containing oil–water emulsions. However, research on the properties of polymer flooding-produced liquids and the behavior of polymers at the oil–water interface remains relatively limited. Therefore, this article will build on existing research to investigate the stability of polymer flooding-produced liquids using molecular dynamics simulations.

## 2. Molecular Model

The S oilfield has adopted polymer flooding technology, specifically using partially hydrolyzed polyacrylamide (HPAM), to enhance oil recovery. At present, molecular simulation has been widely used to study the interfacial properties of surfactant-containing oil–water emulsions [21]. However, research into the properties of polymer flooding liquids and the behavior of polymers at the oil–water interface remains relatively limited. Therefore, this work will build on existing research to investigate the stability of polymer flooding liquids using molecular dynamics simulations. In combination with the actual production, the anions in the mineralized water are mainly Cl^−^, while the cations are dominated by Na^+^ and Ca^2+^ and Mg^2+^. In this study, molecular modelling and molecular dynamics analysis were carried out using Materials Studio software 2020.

### 2.1. Molecular Model of Heavy Oil

**Figure 1:**
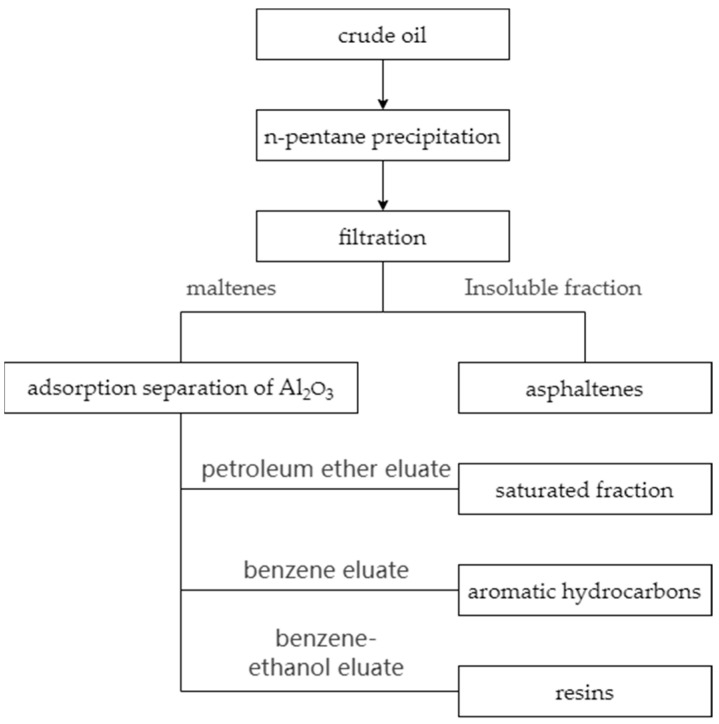
Flow chart for separation of SARA of Oil Product A.

Oil composition, density tests and viscosity tests were conducted on the heavy oil from the S oilfield. The determination of crude oil components adopts the SARA analysis method (saturates, aromatics, resins, and asphaltenes), which classifies the components based on molecular polarity and molecular weight. First, in accordance with the “Test method for separation of asphalt into four fractions” (SH/T 0509-2010) [22], the contents of saturates, aromatics, resins, and asphaltenes in Oil A were determined using liquid–solid adsorption chromatography. The separation flowchart is illustrated in Figure 1.

**Figure 2:**
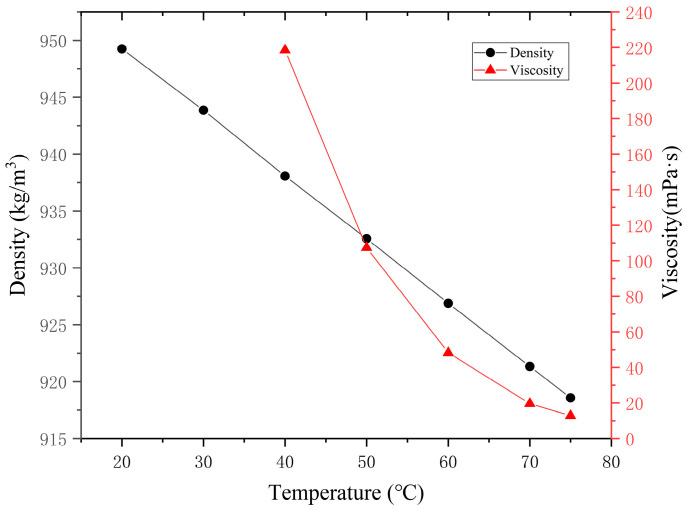
Relationship between density, viscosity and temperature.

In the process of separating the four components of crude oil using liquid–solid chromatography, the asphaltenes were first extracted by a precipitation process according to the ratio of crude oil to n-pentane of 1:30. The extracted asphaltenes were subsequently dried in a vacuum drying oven. For the separated soluble matter, further separation was carried out using a chromatographic column. Al_2_O_3_ was used as the adsorbent for this column. During the rinsing of the column, petroleum ether, benzene, and a 1:1 (*v*/*v*) mixture of benzene and ethanol were used sequentially. At the beginning of the rinsing, petroleum ether was used as the eluent first, and when a light yellow solution was eluted, it was evaporated using a rotary evaporator to obtain the saturated fraction. When the eluate became colorless, the eluent was switched to benzene, and a reddish-brown solution was eluted, which was also processed using the rotary evaporator to obtain the aromatic fraction. When the eluate became colorless again, the eluent was switched to a benzene-ethanol mixture for rinsing until a black solution was eluted, and then the rotary evaporator was used again to obtain the resins. The final test results are shown in Table 1. The density tests and viscosity tests results are shown in Figure 2. These experimental results provide a reliable data foundation for the accurate validation of the heavy oil molecular model.

**Figure 3:**
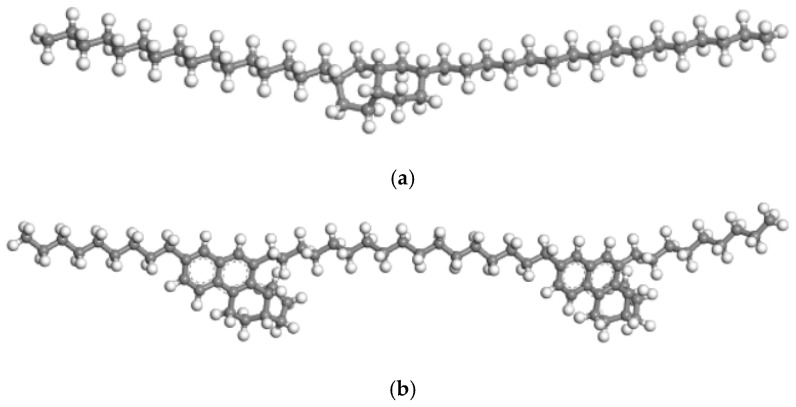

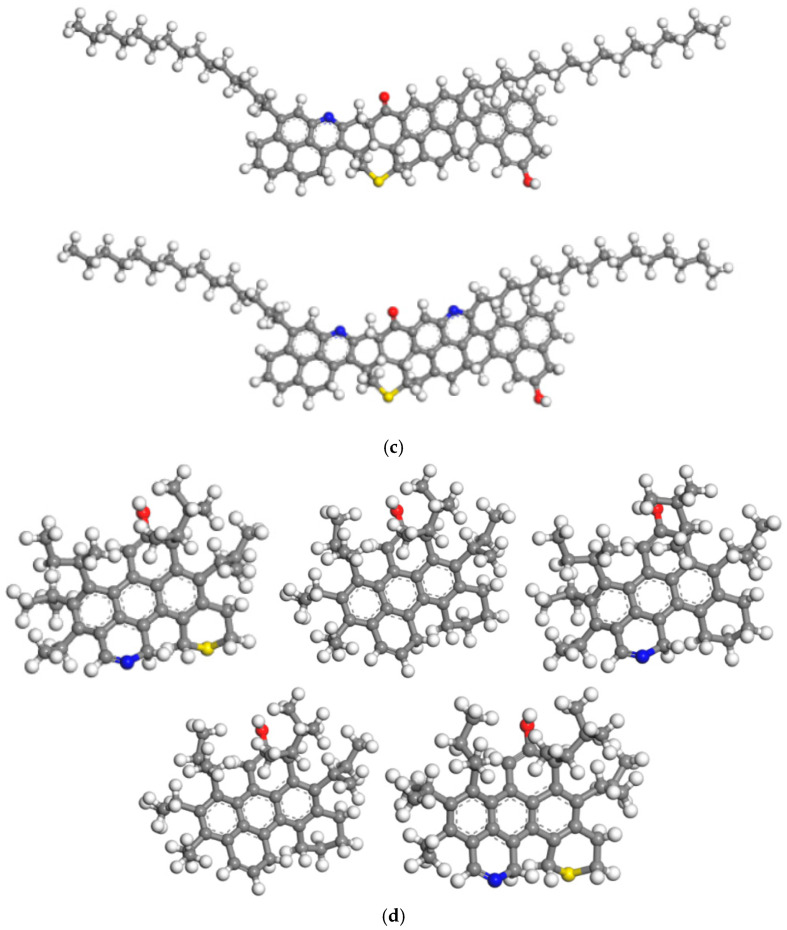
SARA component model (Carbon atoms are represented by gray, hydrogen atoms by white, oxygen atoms by red, nitrogen atoms by blue, and sulfur atoms by yellow): (**a**) SARA saturated fraction model; (**b**) SARA aromatic fraction model; (**c**) SARA resin model; (**d**) SARA asphaltene model.

The structural optimization of heavy oil components from S oilfield was performed based on Yanbin Cao’s [23] analytical results. The procedure was conducted as follows: First, a 3D atomistic document was created in Materials Studio (MS) software, and the molecular models of various heavy oil fractions were constructed using the visualizer module. Prior to molecular dynamics simulations, energy minimization was performed on the initial models through geometry optimization in the Forcite module to eliminate unreasonable atomic contacts in the manually constructed models. The smart algorithm was selected for geometry optimization with the COMPASS III force field. The optimized 3D molecular structures of the four heavy oil fractions are shown in Figure 3, where carbon atoms are represented in gray, hydrogen in white, oxygen in red, nitrogen in blue, and sulfur in yellow. To further minimize the system energy, annealing dynamics was performed after geometry optimization. The simulation was conducted in the NVT ensemble (canonical ensemble with constant number of particles, volume, and temperature) at temperatures cycling between 300 K and 500 K for 1000 ps.

**Figure 4:**
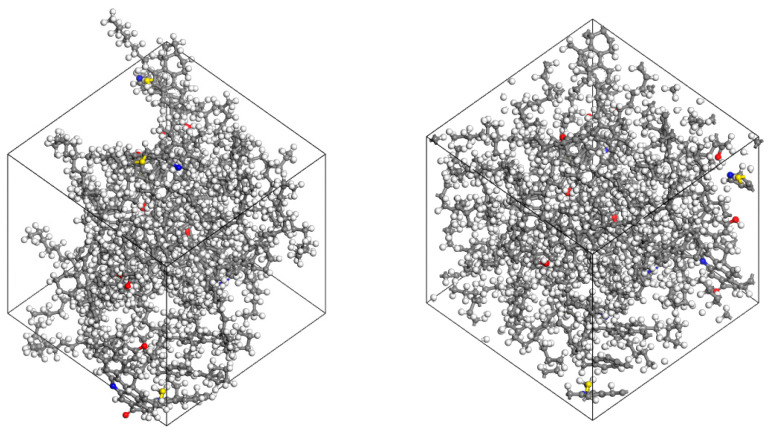
Heavy oil lattice molecules model (Carbon atoms are represented by gray, hydrogen atoms by white, oxygen atoms by red, nitrogen atoms by blue, and sulfur atoms by yellow).

After completing the structural optimization of heavy oil components, the amorphous cell (AC) module was used to establish a simulation lattice with periodic boundary conditions. The component selection was finalized based on the optimized models of the saturated fraction, aromatic fraction, resins, and asphaltenes. The content of each component in the model was determined according to experimental test results, as shown in Table 2. The initial density was set to 1 g/cm^3^, and the COMPASS III force field was applied to obtain the heavy oil molecular model. The structure of the heavy oil molecular model is shown in Figure 4.

**Figure 5:**
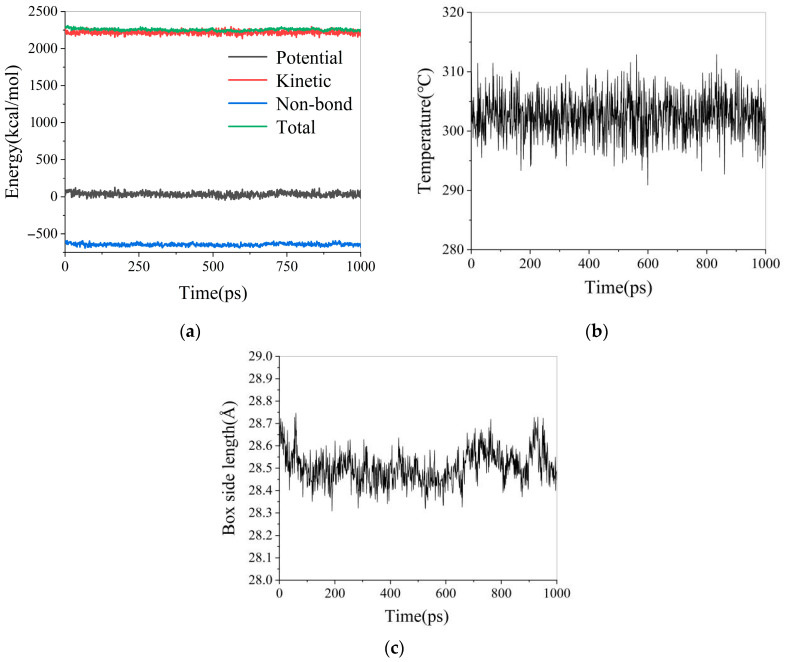
Simulated system energy, temperature, and box size variation diagram: (**a**) energy variation diagram; (**b**) temperature variation diagram; (**c**) box size variation diagram.

**Figure 6:**
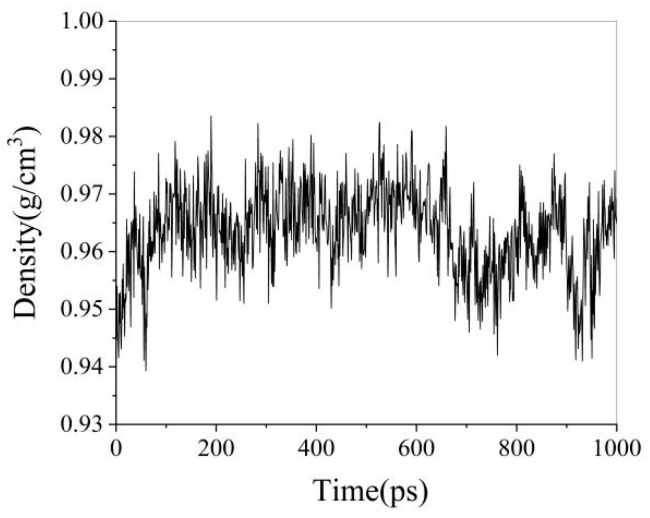
Density variation diagram.

After completing the SARA heavy oil lattice simulation at 303.15 K, the variations in system energy, temperature, and box size over time are shown in Figure 5. The variation in system density over time is shown in Figure 6.

As shown in Figure 5, the temperature and energy profiles of the system exhibit fluctuations as the simulation progresses. With increasing simulation time, the range of these fluctuations gradually decreases and eventually converges to a fixed value. Simultaneously, the box size of the simulated system also stabilizes over time. Therefore, it is concluded that the system reaches equilibrium at a simulation time of 1000 ps.

As shown in Figure 6, the density of the simulated system fluctuates within a small range and stabilizes over time. The last 200 ps of simulation data were extracted to calculate the density of the heavy oil. The average density was determined to be 0.958 g/cm^3^, compared to the experimental measurement of 0.944 g/cm^3^, resulting in a relative error of 1.46%. These results indicate that the constructed heavy oil molecular model aligns well with the actual properties of heavy oil, demonstrating the rationality of the heavy oil box model structure.

### 2.2. Establishment of the Mineralized Water Molecular Model

**Figure 7:**
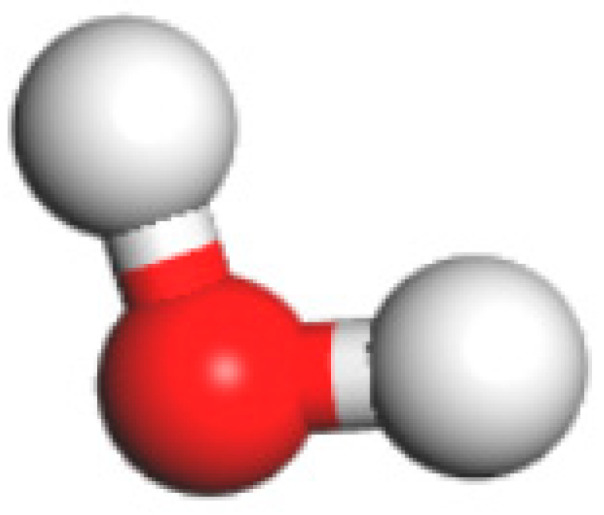
Water molecular model (Oxygen atoms are represented by red, and hydrogen atoms by white).

**Figure 8:**
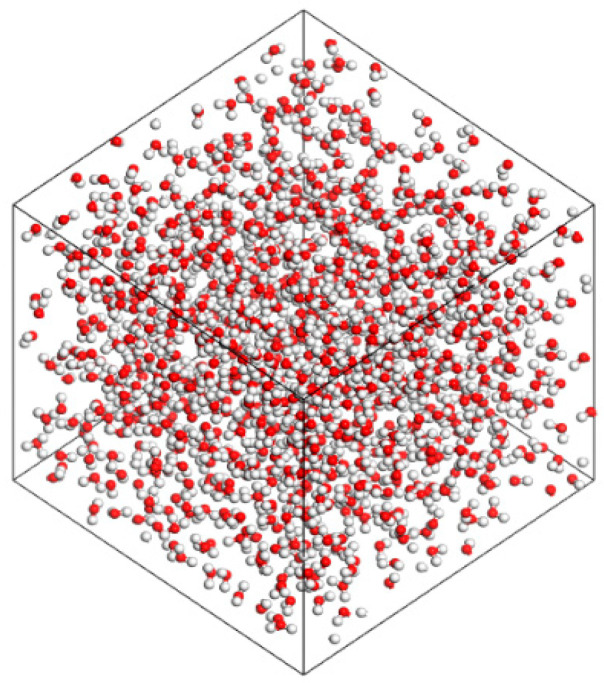
Water box model (Oxygen atoms are represented by red, and hydrogen atoms by white).

The COMPASS III force field was used to generate a water molecule model, as shown in Figure 7. The amorphous cell module was employed to construct a water box containing 1000 water molecules. Forcite was then used to optimize the structure of the water box, resulting in a pure water box model, as shown in Figure 8.

The aqueous phase of oilfield polymer flooding-produced liquids contains various ions. The primary cations are Na^+^, Ca^2+^, and Mg^2+^, while the main anion is Cl^−^.

**Figure 9:**
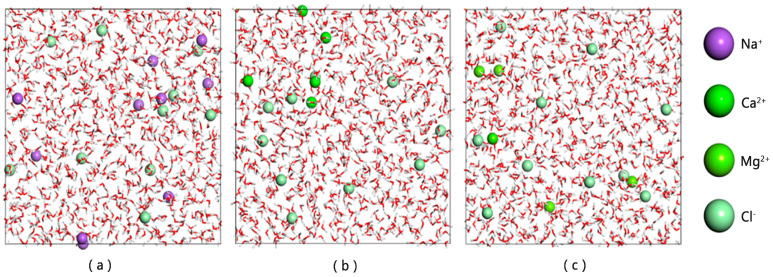
Several water phase models: (**a**) H_2_O + NaCl; (**b**) H_2_O + CaCl_2_; (**c**) H_2_O + MgCl_2_.

NaCl, CaCl_2_ and MgCl_2_ were filled into the water box with anion to cation ratios of [1:1], [1:2] and [1:2], respectively, and the chloride ions were all 10. For clarity, water molecules are represented as stick models, while ions are displayed as ball models and enlarged. The final aqueous phase model, after structural optimization, is shown in Figure 9.

### 2.3. Polymer Molecular Model

**Figure 10:**
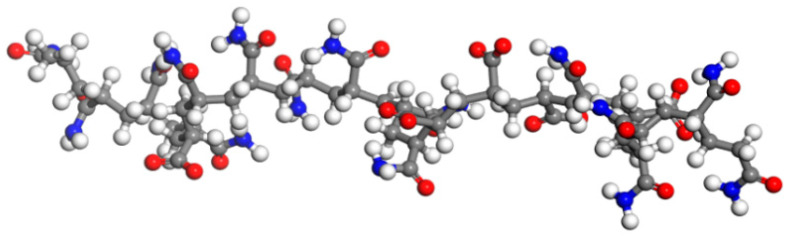
HPAM molecular model (Carbon atoms are represented by gray, hydrogen atoms by white, oxygen atoms by red, and nitrogen atoms by blue).

Currently, the most commonly used polymer for enhanced oil recovery is partially hydrolyzed polyacrylamide (HPAM) [23]. First, considering the actual production conditions of the S oilfield, an HPAM molecular chain with a polymerization degree of 20 and a hydrolysis degree of 25% was constructed. The structure was then optimized using the Forcite module through geometry optimization and anneal dynamics. The optimized polymer molecular model is shown in Figure 10.

**Figure 11:**
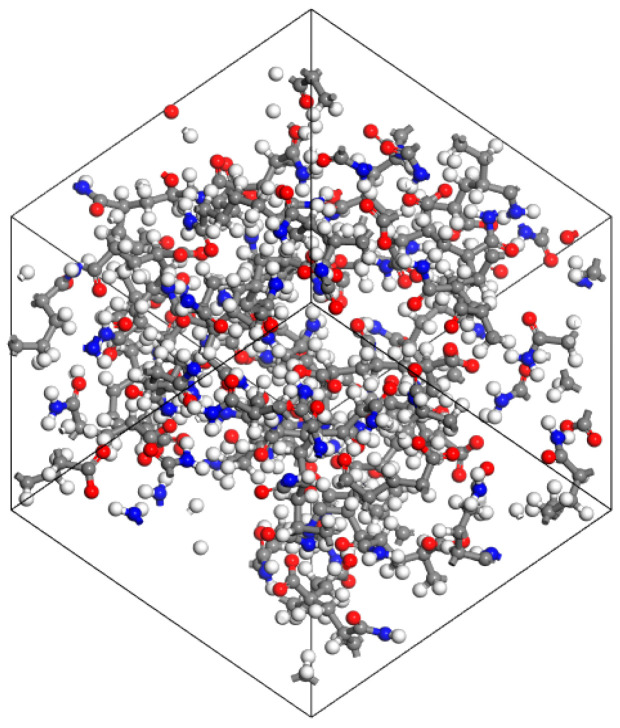
HPAM box (Carbon atoms are represented by gray, hydrogen atoms by white, oxygen atoms by red, and nitrogen atoms by blue).

The HPAM molecular chain was packed into the box. After structural optimization, a complete polymer model was obtained, serving as the foundation for constructing the polymer solution model, as shown in Figure 11.

## 3. Molecular Dynamics Simulation for Stability Analysis of Produced Liquids

### 3.1. Molecular Dynamics Simulation of Oil–Pure Water System

#### 3.1.1. Oil–Pure Water Model Construction

**Figure 12:**
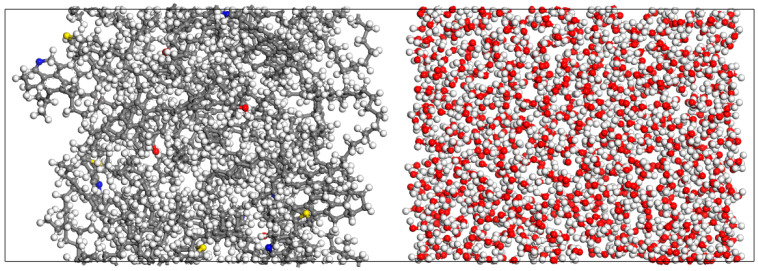
Oil-pure water initial model (Carbon atoms are represented by gray, hydrogen atoms by white, oxygen atoms by red, nitrogen atoms by blue, and sulfur atoms by yellow).

Based on the constructed heavy oil molecular model and water box model, the heavy oil molecules and water box were divided into an oil layer and a water layer, respectively. The build layer module was then used to combine the oil layer and water layer, resulting in the initial configuration of the oil–pure water system, as shown in Figure 12. The initial configuration of the oil–water interface was subjected to structural optimization and molecular dynamics simulations to analyze the movement behavior of molecules at the oil layer surface. Parameters such as interfacial tension and interaction energy, which describe the properties of the oil–water interface, were calculated and analyzed.

#### 3.1.2. Molecular Dynamics Calculation Process

**Figure 13:**
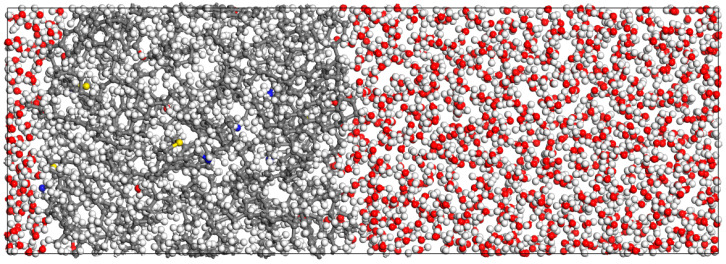
Oil–pure water model(Carbon atoms are represented by gray, hydrogen atoms by white, oxygen atoms by red, nitrogen atoms by blue, and sulfur atoms by yellow).

First, the model underwent structural optimization. In the Forcite module, the dynamics option (molecular dynamics calculation) was selected. The NPT ensemble (constant number of particles, pressure, and temperature) was used, with a temperature of 303.15 K and a pressure of 0.0001 GPa. The COMPASS III force field was applied, and the dynamics calculation was performed for 1000 ps. Subsequently, the NVT ensemble (constant number of particles, volume, and temperature) was selected for further molecular dynamics simulation, also conducted for 1000 ps. The calculation results were output, and the equilibrium state of the oil–pure water system is shown in Figure 13.

**Figure 14:**
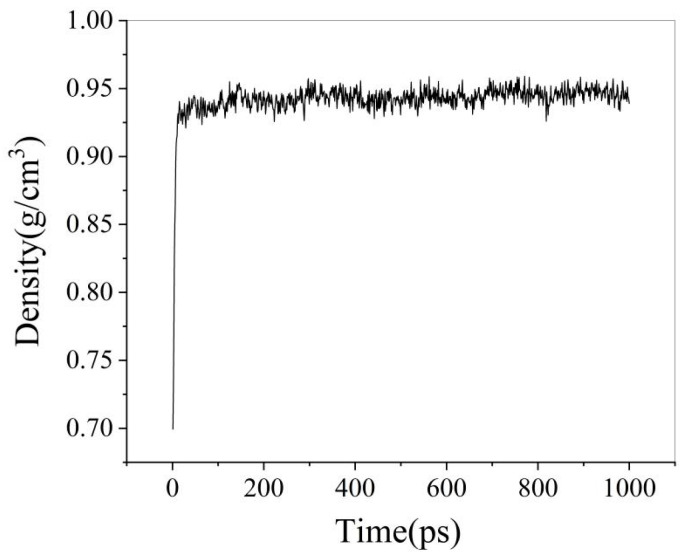
Density variation diagram of oil–pure water model.

After the oil–water system was treated with the NPT ensemble at 303.15 K, the density variation is shown in Figure 14. As illustrated, the density of the system increases rapidly within the first 10 ps. After 10 ps, the density fluctuates within a certain range, indicating that the oil–water system has reached equilibrium.

**Figure 15:**
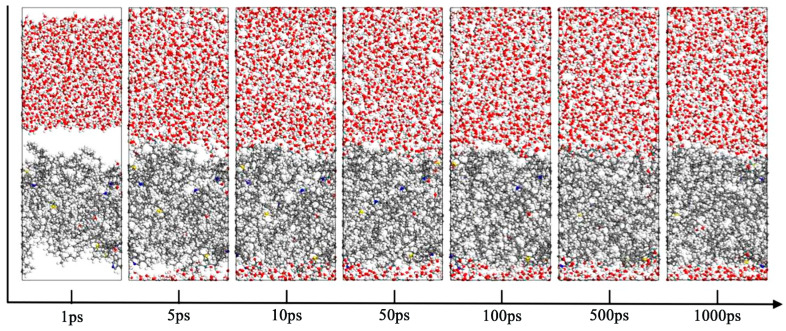
Variation in oil–water interface with time (Carbon atoms are represented by gray, hydrogen atoms by white, oxygen atoms by red, nitrogen atoms by blue, and sulfur atoms by yellow).

To observe molecular behavior changes at the oil–water interface during simulation, snapshots were taken at 1 ps, 5 ps, 10 ps, 50 ps, 100 ps, 500 ps, and 1000 ps, as shown in Figure 15. The most significant molecular changes occurred between the initial state and 10 ps. During this period, water molecules rapidly diffused to the oil surface.

#### 3.1.3. Analysis of Simulation Results

1. Mean square displacement (MSD) and diffusion coefficient of water molecules

**Figure 16:**
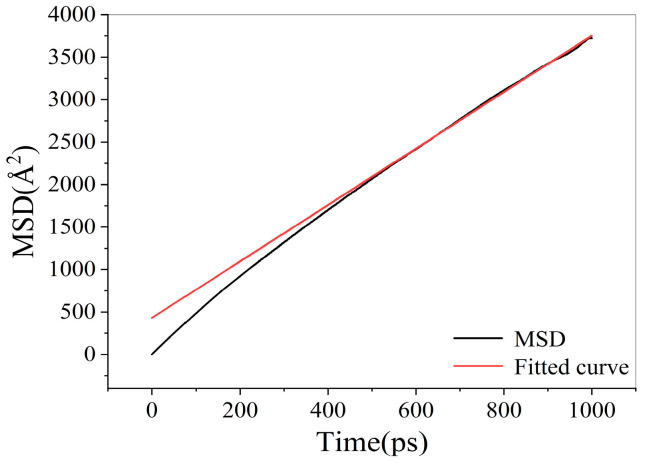
Mean square displacement curve of water molecules.

The mean square displacement (MSD) curve of water molecules is shown in Figure 16. The diffusion coefficient of water molecules in the oil–water system was calculated as 0.6150 Å^2^/ps through MSD data fitting. The fitting coefficient R^2^ is 0.9989, indicating high fitting accuracy.

2. Interfacial tension

The interfacial tension can be calculated by Equation (1)

(1) $$\begin{array}{ccc} \gamma (t) & = & \frac{1}{n}\int_{0}^{L_{z}}\left\{P_{zz}(z,t)-\frac{P_{xx}(z,t)+P_{yy}(z,t)}{2}\right\}dz\\ & = & \frac{L_{z}}{n}\left\{P_{zz}(t)-\frac{P_{xx}(t)+P_{yy}(t)}{2}\right\} \end{array}$$

where *L*_z_ is the length of the simulation box in the z-direction, *P*_xx_, *P*_yy_, and P_zz_ are the pressure components of the pressure in each direction, respectively.

After simulating the system by molecular dynamics simulation under the NVT system, MS software can read the pressure components *P*_xx_, *P*_yy_, *P*_zz_ of the system at each calculation step by Perl script, and the pressure components of each frame of the trajectory are given in the table at the end of the script. The tool obtains the size of the box in each calculation step of the model, obtains the data of *L*_z_ in each frame, averages *P*_xx_, *P*_yy_, *P*_zz_, *L*_z_ can be substituted into Equation (1) to calculate the interfacial stress, and the calculation results are shown in Table 3.

3. Interface interaction energy

Interaction energy serves as a key parameter for evaluating interfacial interaction strength. The interface interaction energy is calculated as shown in Equation (2).

(2) $$E_{\mathrm{int}}=E_{A-B}-\left(E_{A}+E_{B}\right)$$

where *E*_int_ is the interface interaction energy, *E*_A−B_ is the total energy of the two interfaces, *E*_A_ is the energy of interface A alone, *E*_B_ is the energy of interface B alone. The larger the absolute value of the interaction energy, the stronger the interaction between the interfaces.

The Perl script extracted energy values at each timestep (1 ps interval) from Forcite molecular dynamics calculations. The oil–water interfacial interaction energy was computed, with phase A defined as oil and phase B as water. The oil–water interfacial adsorption energy is presented in Table 4 (showing the last 5 ps data). The average interaction energy between oil and pure water at equilibrium was calculated as −126.055 kcal/mol.

### 3.2. Molecular Dynamics Simulation of the Oil–Mineralized Water System

**Figure 17:**
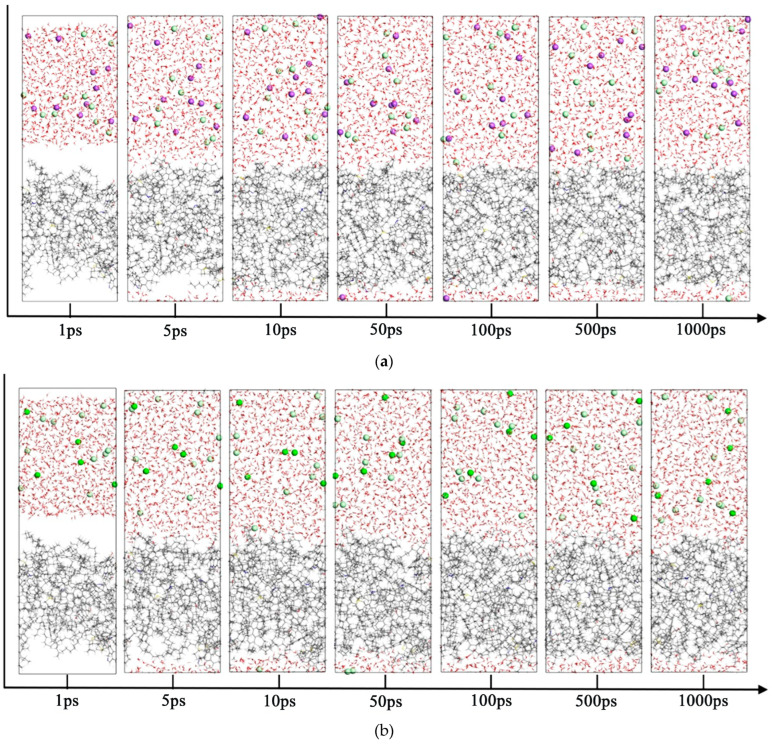

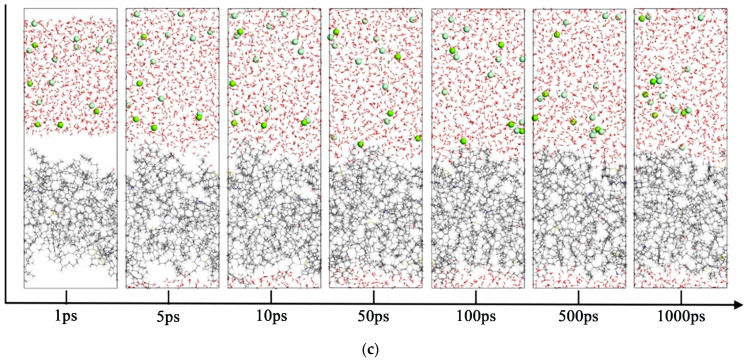
Several systems of oil–water interface variations with time (Carbon atoms are represented by gray, hydrogen atoms by white, oxygen atoms by red, nitrogen atoms by blue, sulfur atoms by yellow, sodium ions by purple, calcium ions by green, magnesium ions by yellow-green, and chloride ions by gray-green): (**a**) Oil-H_2_O-NaCl system; (**b**) Oil-H_2_O-CaCl_2_ system; (**c**) Oil-H_2_O-MgCl_2_ system.

The produced water contains various mineral salts, including NaCl, CaCl_2_, and MgCl_2_. Oil–water systems containing NaCl, CaCl_2_, or MgCl_2_ were modeled and optimized. Molecular dynamics simulations were performed to observe system evolution. Interface snapshots were captured at 1, 5, 10, 50, 100, 500, and 1000 ps. The interfacial evolution is shown in Figure 17.

4. Effect of ions on the diffusion coefficient of water molecules

**Figure 18:**
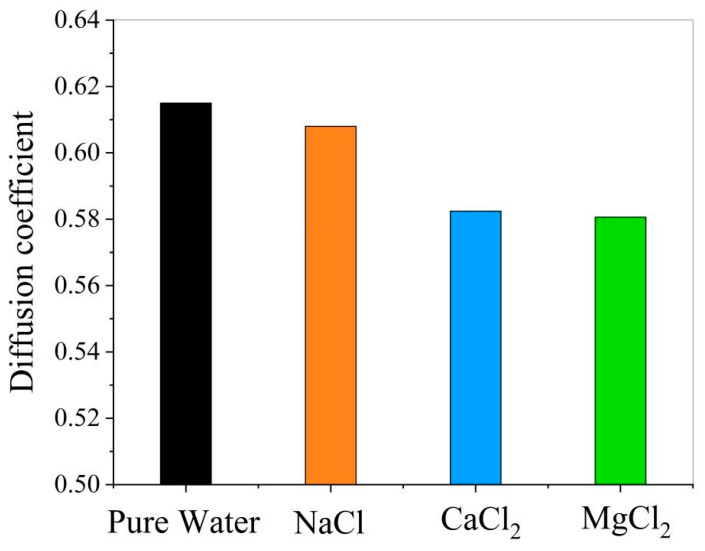
Comparison of diffusion coefficients of water molecules in different oil–water systems.

The influence of ions on water diffusion coefficients was analyzed (Figure 18). The addition of NaCl, CaCl_2_, or MgCl_2_ to the oil–pure water system reduced water diffusion coefficients. This indicates decreased water mobility and enhanced interfacial stability.

5. Comparison of radial distribution of ions in different mineralized water systems

The radial distribution function (RDF) is an important parameter for characterizing the structure of emulsions [24]. As a basic function in the study of liquid or disordered systems, the radial distribution function, *g*_ij_(*r*), is used to describe the average number of *j* atoms per unit volume at distance *r* from the target atom *i* [25]. Its calculation equations are Equations (3) and (4).

(3) $$g_{ij}\left(r\right)=\frac{dN}{4\pi r^{2}\rho dr}$$

(4) $$dN=4\pi r^{2}\rho g_{ij}(r)dr$$

where *g_i_*_j_(*r*) is the radial distribution function, *r* is the calculated radius, *dN* is the number of target atoms between radius *r* and *r + dr*, *ρ* is the atomic density.

**Figure 19:**
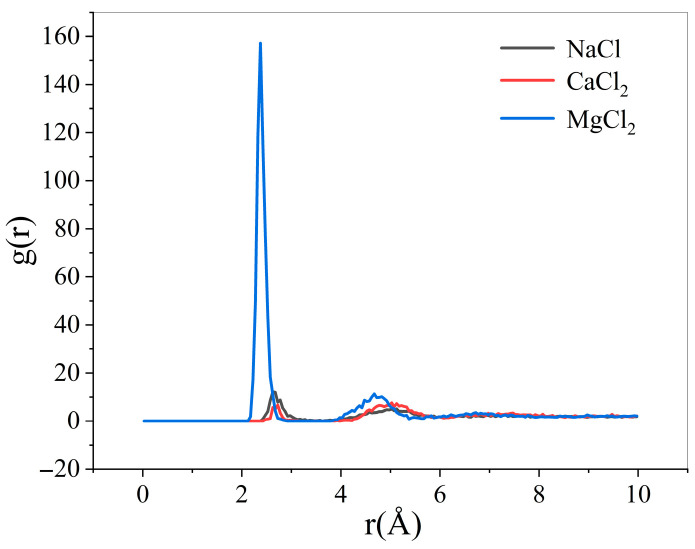
Comparison of radial distribution of ions in different mineralized water systems.

Cl^−^ in water was selected as the target atom, and the average number of Na^+^, Mg^2+^, and Ca^2+^ with a distance of 10 Å from Cl^−^ per unit volume was analyzed to calculate the radial distribution function between Cl^−^ and Na^+^, Mg^2+^, and Ca^2+^. Figure 19 compares the ion radial distributions in different mineralized water systems. All systems exhibited RDF peaks at 2~3 Å radius. The MgCl_2_-containing system showed the highest RDF peak intensity, indicating the strongest ion interactions.

6. Effect of ions on the interfacial tension of oil and water

**Figure 20:**
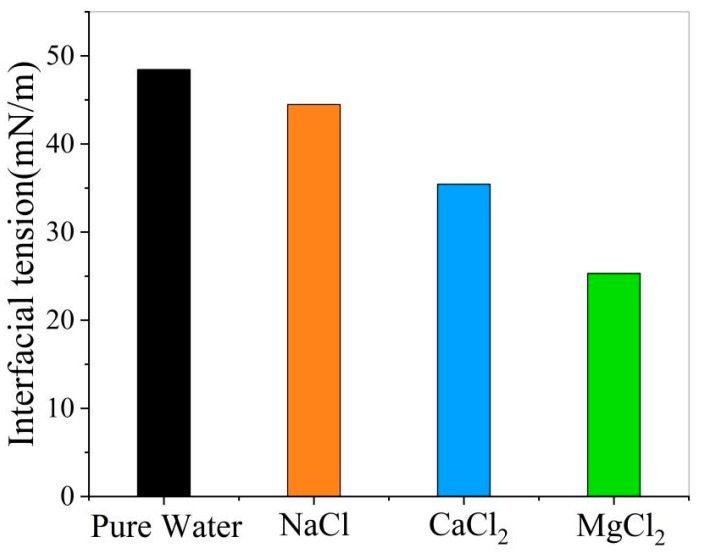
The effect of ions on the interfacial tension between oil and water.

Figure 20 demonstrates that adding NaCl, CaCl_2_, or MgCl_2_ to the oil–pure water system reduces interfacial tension in all cases. This indicates enhanced emulsion stability at the oil–water interface in the presence of ions.

7. Effect of ions on interfacial interaction energy

**Figure 21:**
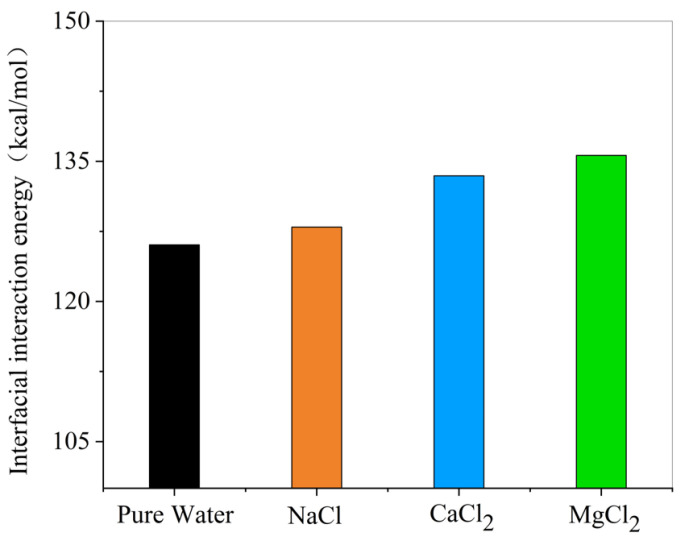
Influence of ions on interfacial interaction energy.

As shown in Figure 21, the absolute value of oil–water interfacial interaction energy increases with the addition of NaCl, CaCl_2_, or MgCl_2_. This indicates enhanced interfacial interactions due to the ions.

Within the analyzed ion concentration range, ions stabilized the oil–water interface compared to pure water. The stabilization effectiveness followed the order: Mg^2+^ > Ca^2+^ > Na^+^ at equivalent cation concentrations. Analysis of the water molecular diffusion coefficients at the interface demonstrated that ionic additives reduced interfacial tension, thereby decreasing water mobility and enhancing interface stability.

### 3.3. Molecular Dynamics Simulation of Oil–Polymer–Mineralized Water System

**Figure 22:**
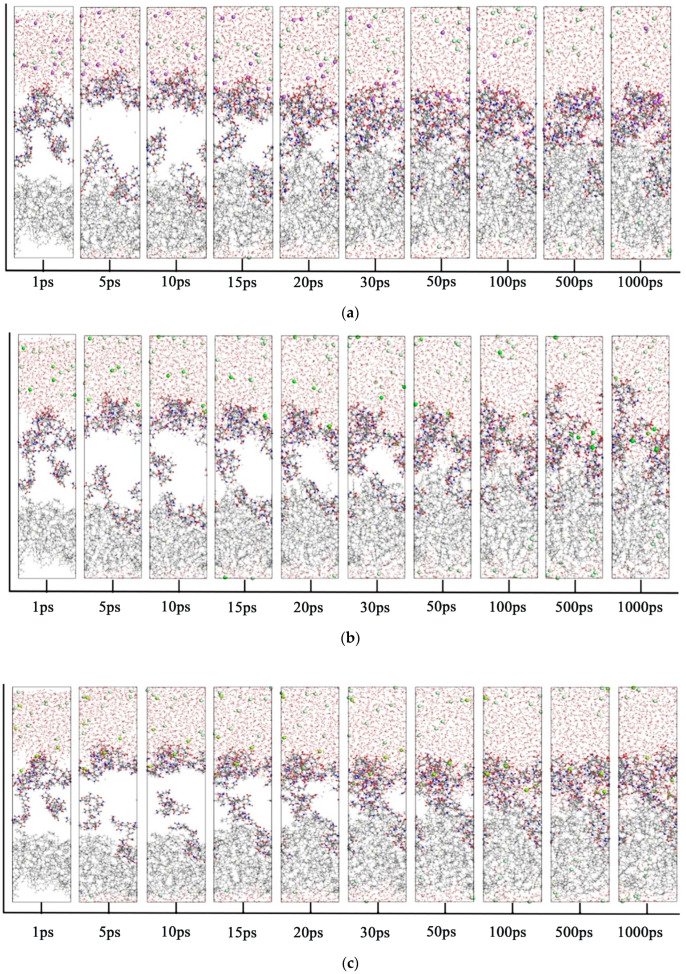
Time-dependent molecular behavior of different systems (Carbon atoms are represented by gray, hydrogen atoms by white, oxygen atoms by red, nitrogen atoms by blue, sulfur atoms by yellow, sodium ions by purple, calcium ions by green, magnesium ions by yellow-green, and chloride ions by gray-green): (**a**) Oil-HPAM-H_2_O-NaCl system; (**b**) Oil-HPAM-H_2_O-CaCl_2_ system; (**c**) Oil-HPAM-H_2_O-MgCl_2_ system.

The initial configuration of the oil–polymer–mineralized water system was constructed using the build layer module, based on pre-established heavy oil, polymer, and water box models. This configuration enabled the analysis of polymer effects on oil–water interfacial parameters. The initial oil–water interface configuration was structurally optimized. Molecular dynamics simulations were performed using the Forcite dynamics module with NPT ensemble and COMPASS III force field (303.15 K, 1000 ps) to obtain diffusion parameters including mean square displacement (MSD) and radial distribution function (RDF). Additional simulations using NVT ensemble and COMPASS III force field (303.15 K, 1000 ps) provided interfacial interaction energy and tension data. The system underwent further structural optimization with Forcite, followed by final NPT ensemble molecular dynamics simulations (303.15 K, 1000 ps). The oil–water interface images corresponding to 1 ps, 5 ps, 10 ps, 15 ps, 20 ps, 30 ps, 50 ps, 100 ps, 500 ps and 1000 ps were captured and the changes are shown in Figure 22. The changes in the behavior of the various substances in the system are well illustrated. It was observed that HPAM showed strong hydrophilicity, and due to the anionic groups on the molecular chain, the Na^+^ ions in water, some Ca^2+^ ions in water and Mg^2+^ ions in water were gradually attracted and approached the HPAM. As the simulation progressed, the HPAM gradually aggregated at the oil–water interface.

8. Effect of polymers on the radial distribution of cations in water

**Figure 23:**
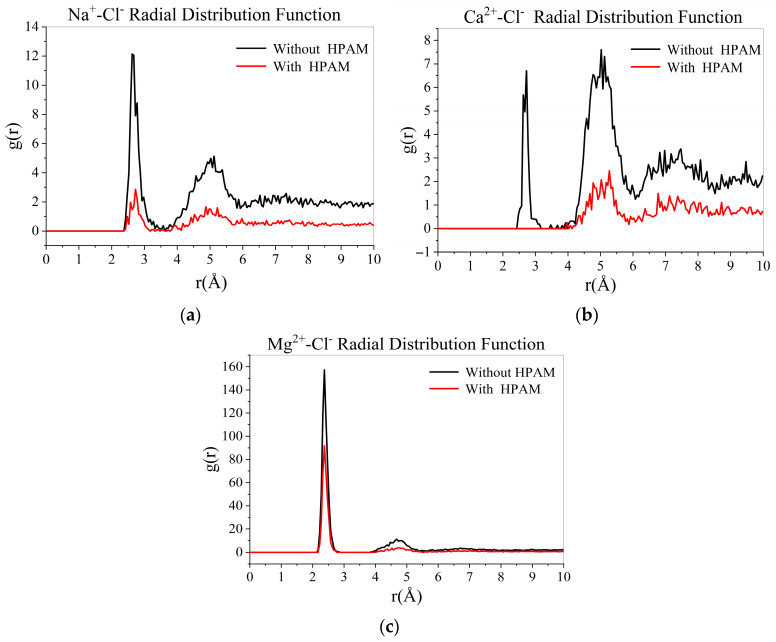
Effect of HPAM on the radial distribution of several cations: (**a**) Na^+^-Cl^−^ RDF; (**b**) Ca^2+^-Cl^−^ RDF; (**c**) Mg^2+^-Cl^−^ RDF.

Figure 23 shows HPAM’s effect on cation radial distribution. The addition of HPAM reduced the radial distribution function (RDF) values for both Cl⁻ and cations in the aqueous phase. This reduction likely results from the electrostatic attraction between negatively charged HPAM hydrolysis groups and cations. As evidenced in Figure 23, pronounced peaks appeared in the RDF curves of HPAM-cation pairs, confirming their strong interaction.

9. Effect of polymers on oil–water interfacial tension

**Figure 24:**
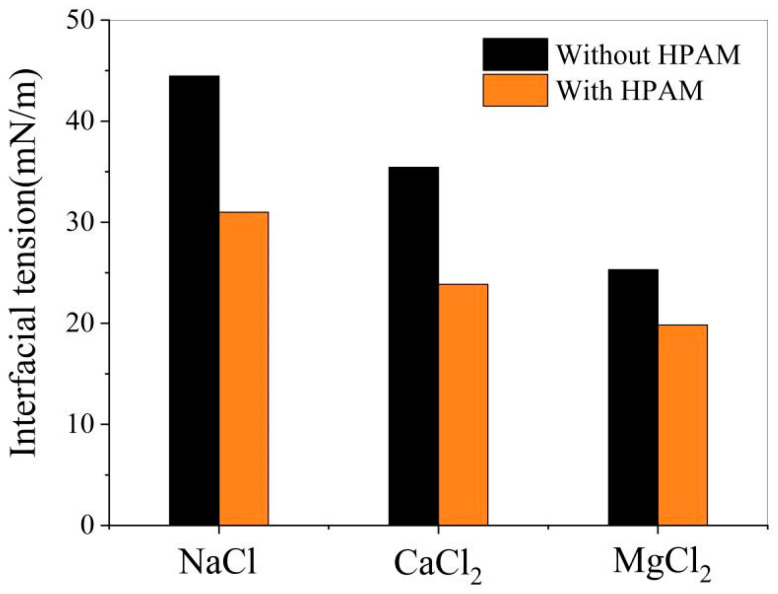
Effect of HPAM on oil–water interfacial tension.

Figure 24 shows that adding HPAM reduces interfacial tension in all three oil–water systems. This demonstrates enhanced emulsion stability with polymer presence.

10. Effect of polymers on interfacial interaction energy

**Figure 25:**
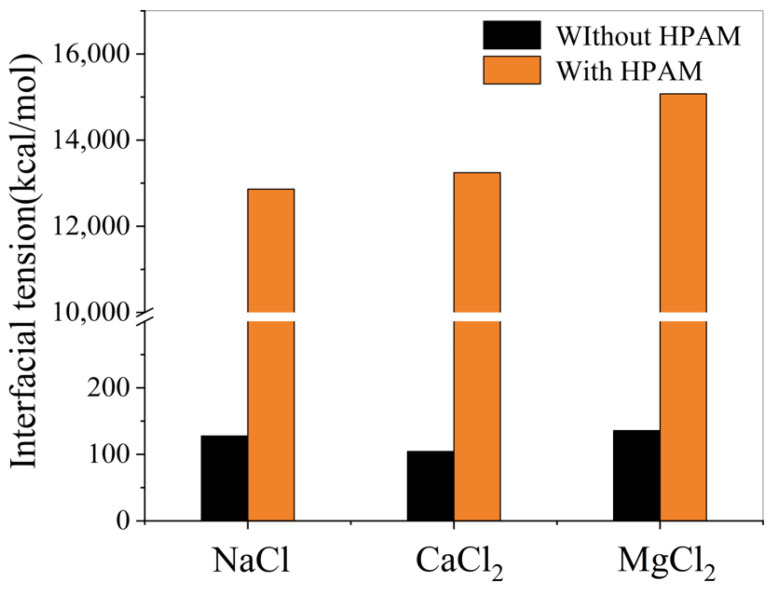
Effect of HPAM on the interaction energy of oil–water interface.

Based on the simulation data and Figure 25, the addition of HPAM polymer significantly increases the absolute value of the oil–water interfacial energy of interaction. This demonstrates the ability of HPAM to improve interfacial interactions.

## 4. Conclusions

1. In molecular modeling, fundamental models of heavy oil, water and polymers were successfully established. The accuracy of these molecular models was verified through comparative analysis between experimental measurements and simulation results. Specifically, the experimental density of heavy oil was determined to be 0.944 g/cm^3^, while the simulated value yielded 0.958 g/cm^3^, showing a relative error of only 1.46%. For the water box model, the actual ionic composition of field water samples was incorporated, with particular emphasis on investigating the interfacial effects of cations such as Na^+^, Ca^2+^, and Mg^2+^ at oil–water interfaces.
2. The presence of ions (NaCl, CaCl_2_, MgCl_2_) reduces oil–water interfacial tension and enhances interfacial interactions, thereby improving emulsion stability. The degree to which cations reduce the interfacial tension between oil and water is Mg^2+^ > Ca^2+^ > Na^+^.
3. In the simulation of the oil/polymer/water system, it was observed that the cations in the water attract the anionic groups hydrolyzed from the HPAM polymer chains, thereby enhancing the hydrophilicity of the HPAM polymer. Meanwhile, the lipophilic segments of the polymer dissolve into the heavy oil, forming an interfacial film at the oil–water interface. This film reduces the interfacial tension of the emulsion and improves its stability.

## Figures and Tables

**Table 1:** Content of SARA of oil product A.

Composition	Mass Fraction (%)
Saturated fraction	26.6
Aromatic hydrocarbons	35.2
Resins	18.1
Asphaltenes	20.1

**Table 2:** Component content of heavy oil model.

Composition	Quantity	Mass Fraction (%)
Saturates	6	26.22
Aromatics	5	34.74
Asphaltenes1	1	4.44
Asphaltenes2	1	4.29
Asphaltenes3	1	4.30
Asphaltenes4	1	4.29
Asphaltenes5	1	4.44
Resins1	1	8.64
Resins2	1	8.65

**Table 3:** Oil–pure water interfacial tension.

*P*_xx_ (GPa)	*P*_yy_ (GPa)	*P*_zz_ (GPa)	*L*_z_ (Å)	Interfacial Tension (mN/m)
−0.036	−0.037	−0.031	84.721	48.439

**Table 4:** Oil–pure water interface interaction energy.

*E_A−B_* (kcal/mol)	*E_A_* (kcal/mol)	*E_B_* (kcal/mol)	*E*_int_ (kcal/mol)
−7689.527	206.521	−7769.993	−126.055

## Data Availability

The original contributions presented in this study are included in the article. Further inquiries can be directed to the corresponding author.
